# A multi-chip integrated analysis employing machine learning techniques identifies biomarkers for diagnosing PCOS and its comorbidity with T2D

**DOI:** 10.1097/MS9.0000000000005168

**Published:** 2026-06-19

**Authors:** Shigui Tu

**Affiliations:** Department of Laboratory Medicine, The First Affiliated Hospital of Chongqing Medical University, Chongqing, China

**Keywords:** bioinformatics, gene chip, machine learning, polycystic ovary syndrome, type 2 diabetes

## Abstract

Polycystic ovary syndrome (PCOS) is a common endocrine disorder closely linked to type 2 diabetes (T2D). Although linked, potential biomarkers for PCOS have not yet been sufficiently studied. This study aimed to identify diagnostic biomarkers linked to PCOS through an integrative analysis of multi-chip data and to examine their relationship with T2D. We used multi-chip datasets from public databases, conducted differential expression analysis to screen genes, and performed cross-disease gene overlap analysis using Venn diagrams. We conducted functional enrichment analyses, including gene ontology and Kyoto Encyclopedia of Genes and Genomes analyses, to investigate the biological significance of the intersecting genes. Based on these findings, we developed a protein-protein interaction network and identified key hub genes. We utilized a Least Absolute Shrinkage and Selection Operator regression model with 10-fold cross-validation for modeling. Subsequently, univariate and multivariate logistic regression analyses were performed to assess T2D risk. We also assessed the model’s ability to distinguish T2D using a diagnostic nomogram. Our results identified PRPF31, HABP4, CTPS2, and GFM1 as core hub genes with diagnostic potential. The effectiveness of these biomarkers was validated using receiver operating characteristic curve analysis across multiple datasets. Logistic regression analyses revealed that PRPF31 serves as an independent predictor of T2D, with reduced expression significantly correlating with increased T2D risk. The C-index from the diagnostic nomogram analysis indicated that the model possessed a strong discriminative ability for T2D. These findings enhance our understanding of the molecular mechanisms linking PCOS and T2D, laying the groundwork for clinical applications in the early detection and management of PCOS and its associated conditions.

## Introduction

Polycystic ovary syndrome (PCOS) is a common endocrine disorder in women of reproductive age and is characterized by ovarian dysfunction, high androgen levels, and polycystic ovary morphology. Irregular menstrual cycles, weight gain, and insulin resistance are common symptoms^[^1–3^]^. These symptoms can significantly impact a woman’s quality of life. Epidemiological research indicates that around 10% of women of childbearing age have PCOS, which is closely linked to obesity, insulin resistance, and a heightened risk of type 2 diabetes (T2D), underscoring the interrelation of these factors^[^4–6^]^. Despite its prevalence, the diagnostic criteria for PCOS remain contentious due to differences in definitions and thresholds among various guidelines; for instance, the Rotterdam criteria expand upon the original NIH criteria by incorporating additional clinical manifestations. This expansion may complicate the accurate identification of the condition and the management of its long-term risks^[^7,8^]^. Consequently, it is crucial to explore the underlying endocrine and metabolic mechanisms of PCOS and to identify reliable biomarkers that can enhance diagnostic precision and inform effective treatment strategies.

HIGHLIGHTS

- First, traditional single-omics methods fall short; we need to integrate multi-omics to explore clinical applications.
- Second, by combining integrated multi-omics analysis with Least Absolute Shrinkage and Selection Operator regression, we can help identify potential biomarkers for polycystic ovary syndrome (PCOS). This approach allows for more accurate identification.
- Third, finding potential biomarkers in PCOS patients sheds more light on their risk of developing type 2 diabetes.

T2D has been steadily increasing worldwide, reflecting a concerning trend in global health[9]. This complex and progressive condition is primarily characterized by inadequate insulin production from the β-cells of the pancreas, which are not affected by autoimmunity. It is often accompanied by insulin resistance and metabolic syndrome^[^10,11^]^. T2D constitutes 96% of all diabetes cases and is intricately associated with a range of metabolic disorders. It is a major noncommunicable chronic disease that poses a significant threat to human health. Despite its prevalence, our understanding of the mechanisms underlying its development remains incomplete[12].

Studies have shown that adolescent girls with PCOS have an increased risk of T2D, with about one-third displaying metabolic abnormalities^[^13,14^]^. This link arises from a common underlying mechanism, as insulin resistance is pivotal in both conditions[15]. Although there has been a growing number of studies examining the relationship between PCOS and T2D, a significant gap in knowledge remains in comprehensive multi-chip analyses. Many investigations have focused on the link between these two disorders; however, the specific molecular mechanisms and potential biomarkers that underlie this connection are not well understood. This lack of clarity complicates our understanding of how PCOS and T2D are related, thereby posing challenges for accurate diagnosis and effective treatment. Additionally, traditional single-omics research methods struggle to capture the intricate biological processes. These processes involve multi-level regulation of genes, proteins, metabolites, and other factors. Therefore, it is essential to adopt integrated multi-chip analysis methods to identify potential biomarkers more effectively and further explore their clinical applications.

Integrating data from genomics, transcriptomics, and proteomics has emerged as a powerful research strategy in the fast-evolving field of bioinformatics. This approach allows the collection of biological information at different molecular and cellular levels. Such comprehensive data uncover the fundamental mechanisms of diseases and identify potential biomarkers[16]. The ChatGPT model, based on machine learning algorithms, is increasingly applied in the medical field, showing a good development trend[17]. By integrating these datasets and employing machine learning techniques, researchers can extract meaningful insights from intricate data. This valuable information will enable the development of predictive models for early disease diagnosis, ultimately enhancing diagnostic accuracy and improving treatment outcomes.

This study sought to identify biomarkers linked to PCOS through bioinformatics analysis and to investigate how these PCOS-related biomarkers are connected to T2D, thereby uncovering the mechanisms underlying their co-occurrence. Furthermore, the research employed machine learning techniques to develop a robust diagnostic model based on these biomarkers, aiming to provide clinicians with more precise and tailored diagnostic and treatment options. This approach is anticipated to significantly contribute to the advancement of personalized medicine.

This study applies machine learning techniques by utilizing the Least Absolute Shrinkage and Selection Operator (LASSO) regression to identify biomarkers linked to PCOS that comply with the TITAN guidelines[18].

## Methods

### Data sources

Four microarray datasets were obtained from the Gene Expression Omnibus (GEO) database (https://www.ncbi.nlm.nih.gov/geo/): GSE155489 (GPL20795), GSE34526 (GPL570), GSE54248 (GPL10558), and GSE184050 (GPL11154). The GSE155489 dataset included 20 samples, comprising 12 oocyte samples, with six from PCOS patients and six from control subjects. Additionally, eight samples were obtained from follicular granulosa cells, equally divided between four PCOS patients and four controls. The GSE34526 dataset consisted of 10 samples, all from follicular granulosa cells, with seven from PCOS patients and three from controls. The GSE54248 dataset contained eight samples, all collected from peripheral blood, evenly split, with four from PCOS patients and four from controls. Finally, the GSE184050 dataset included 116 samples, also from peripheral blood. After excluding 20 male samples, we retained 96 female samples, which consisted of 40 patients with T2D and 56 controls. After downloading either the microarray expression matrix files or the normalized count transcripts per million (TPM) data files for each dataset, we used GEO2R to analyze the datasets and extract gene expression information.

### Methods

#### Gene matrix annotation

Datasets GSE155489, GSE34526, GSE54248, and GSE184050 were processed by organizing and analyzing the data using Excel in Microsoft Office Home and Student 2016 (version 16.0.19127.20264). Subsequently, gene names obtained from the gene sequence files analyzed with GEO2R were used to update the gene identifiers in the original matrix files or normalized (norm count TPM) data files. This ensured that each gene identifier accurately corresponded to its respective gene name.

#### Merging, batch correction, and heterogeneity analysis across different tissue types of the PCOS dataset multi-chip matrices

Initially, we merged the expression data matrices from the GSE34526 and GSE155489 datasets by aligning and concatenating them based on common gene identifiers using Excel functions in Microsoft Office Home and Student 2016 (version 16.0.19127.20264). Batch correction was performed using the sva package (version 3.44.0) in R (version 3.6.2). This step is essential for correcting batch effects and other systematic biases in the data, resulting in a batch-corrected and normalized expression matrix. The combined dataset includes two different tissue types: granulosa cells and oocytes. To analyze the heterogeneity between these tissue types, the variancePartition package (version 1.10.1) was used. *R*^2^ (coefficient of determination) represents the percentage of gene expression variation that can be independently explained by tissue type, and the smaller this value, the less gene expression variability is attributable to tissue type. An *R*^2^ value less than 0.1 indicates that gene expression differences are minimally influenced by tissue type.

#### Differential analysis

This study conducted differential expression analysis on gene expression matrices derived from PCOS samples. We utilized the limma package (version 3.52.2) in R (version 4.2.1) to integrate multiple datasets. We applied screening criteria to identify differentially expressed genes (DEGs), selecting those with an absolute |log2 Fold Change (FC)| > 0.58 and a *P*-value < 0.05. We used the ComplexHeatmap package (version 2.13.1) to generate a heatmap and the ggplot2 package (version 3.4.4) to create a volcano plot for visualizing the DEGs.

#### Identification of genes associated with T2D in PCOS patients

Candidate genes were initially identified from the GSE184050 dataset using a screening threshold of *P* < 0.05. Following this, we conducted an overlap analysis between these candidate genes and previously identified DEGs. The overlapping genes from this analysis were considered the final candidate genes. To visualize the results of this overlap analysis, we used the ggplot2 package (version 3.4.4) and the VennDiagram package (version 1.7.3) in R (version 4.2.1).

#### Gene function annotation and signal pathway analysis

Functional enrichment analysis of candidate genes, selected by log2 FC, was conducted using the R packages clusterProfiler (version 4.4.4) and GOplot (version 1.0.2). This analysis focused on their associations with the gene ontology (GO) and Kyoto Encyclopedia of Genes and Genomes (KEGG) pathways. The ggplot2 package (version 3.4.4) in R (version 4.2.1) was used to visualize the enrichment analysis results.

#### Construction of protein-protein interaction network and identification of hub genes

We utilized the STRING database (https://string-db.org/) to analyze the interactions among proteins encoded by the candidate genes. After importing the protein-protein interaction (PPI) network data into Cytoscape (version 3.9.1), we used the CytoHubba plugin to identify the top 10 hub genes based on three algorithms: Maximal Clique Centrality (MCC), Degree, and Edge Percolated Component (EPC). We then performed an intersection analysis of the genes identified using these methods to determine the core hub genes. This intersection analysis was performed using (specified method or software, if applicable). Finally, R (version 4.2.1) and the ggplot2 package (version 3.4.4) were used to create a Venn diagram that visualizes the overlap among the gene sets from the intersection analysis.

#### LASSO logistic regression analysis

The LASSO logistic regression method, utilizing 10-fold cross-validation, was applied to analyze hub genes. This method was used to identify genes significantly linked to PCOS, and genes with *R*^2^ < 0.1 in the tissue heterogeneity analysis were identified as final biomarkers for PCOS patients.

#### Analysis and validation of biomarker gene diagnostic performance in PCOS patients

To perform receiver operating characteristic (ROC) analysis with the GSE54248 dataset, we first acquired a combined dataset containing merged samples from various sources and the expression levels of biomarker genes for each sample, which typically involves downloading the dataset from the GEO database and processing it using R (version 4.2.1). Once the dataset is prepared, the pROC package (version 1.18.0) in R (version 4.2.1) is used for ROC analysis. The area under the ROC curve (AUC) will help to evaluate the diagnostic accuracy of the biomarker genes. An AUC of 0.5 signifies no diagnostic discrimination, whereas an AUC of 0.5–0.7 indicates low diagnostic accuracy. An AUC between 0.7 and 0.9 indicates moderate diagnostic accuracy, while an AUC > 0.9 indicates high diagnostic accuracy. ROC analysis results can be visualized using the ggplot2 package (version 3.4.4) in R (version 4.2.1). This will allow for the creation of informative plots that effectively represent the diagnostic capabilities of biomarker genes.

#### Univariate and multivariate logistic regression analyses, as well as diagnostic nomogram analysis

Initially, the expression levels of candidate biomarkers were extracted from each sample within the GSE184050 dataset. Univariate and multivariate logistic regression analyses were performed using the rms package (version 6.4.0) and the Resource Selection package (version 0.3–5) in R (version 4.2.1). Regression results were visualized using a forest plot created with the ggplot2 package (version 3.4.4). A diagnostic nomogram analysis was conducted using the rms package (version 6.4.0), and the results were visualized.

#### Methodological statement

The report of this study adheres to the STARD (Standards for Reporting Diagnostic Accuracy Studies) guidelines.

## Results

### Identifying DEGs in PCOS patients

**Figure 1.**
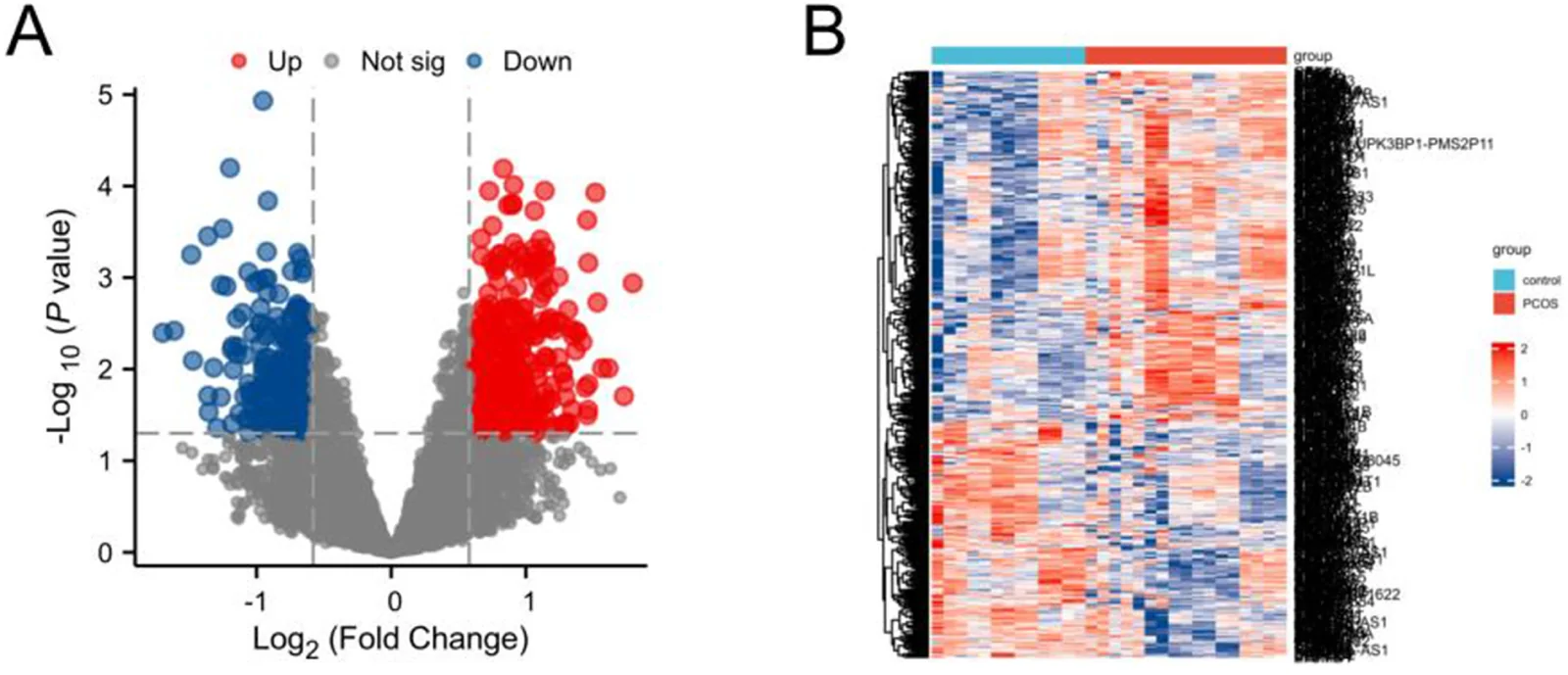
(A) A volcano plot illustrates DEGs from the combined datasets GSE34526 and GSE155489, highlighting 351 upregulated and 236 downregulated genes. (B) A heatmap of DEGs from the same merged datasets.

This study identified 587 DEGs across various datasets of patients with PCOS. A total of 351 genes were upregulated, and 236 genes were downregulated compared to those in the control group (Fig. 1A and 1B).

### Intersection analysis of DEGs in PCOS with target genes in T2D and screening for candidate genes

**Figure 2.**
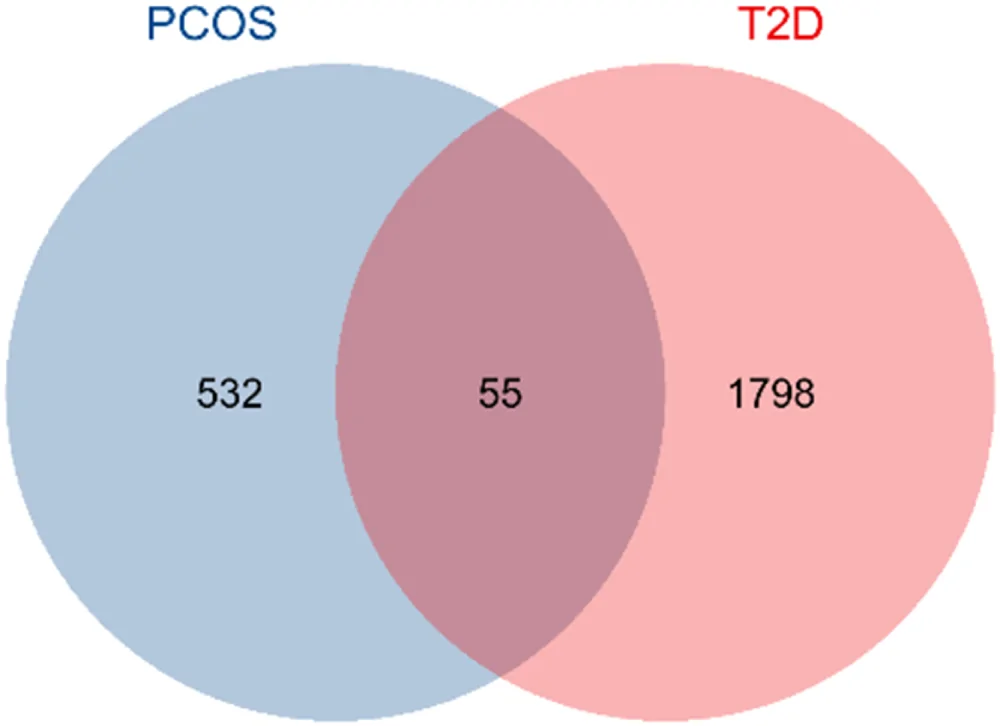
Presents a Venn diagram illustrating the intersection of differentially expressed genes (DEGs) in PCOS with candidate genes linked to T2D.

Analysis of the gene expression profiles from the GSE184050 dataset identified 1853 genes associated with T2D that met the screening criterion of *P*-value < 0.05. To identify candidate genes relevant to both PCOS and T2D, we used a Venn diagram to examine the overlap between T2D-associated genes and DEGs identified in PCOS. This analysis identified 55 candidate genes (Fig. 2).

### Enrichment analysis of candidate genes GO and KEGG combined with Log2 FC

**Figure 3.**
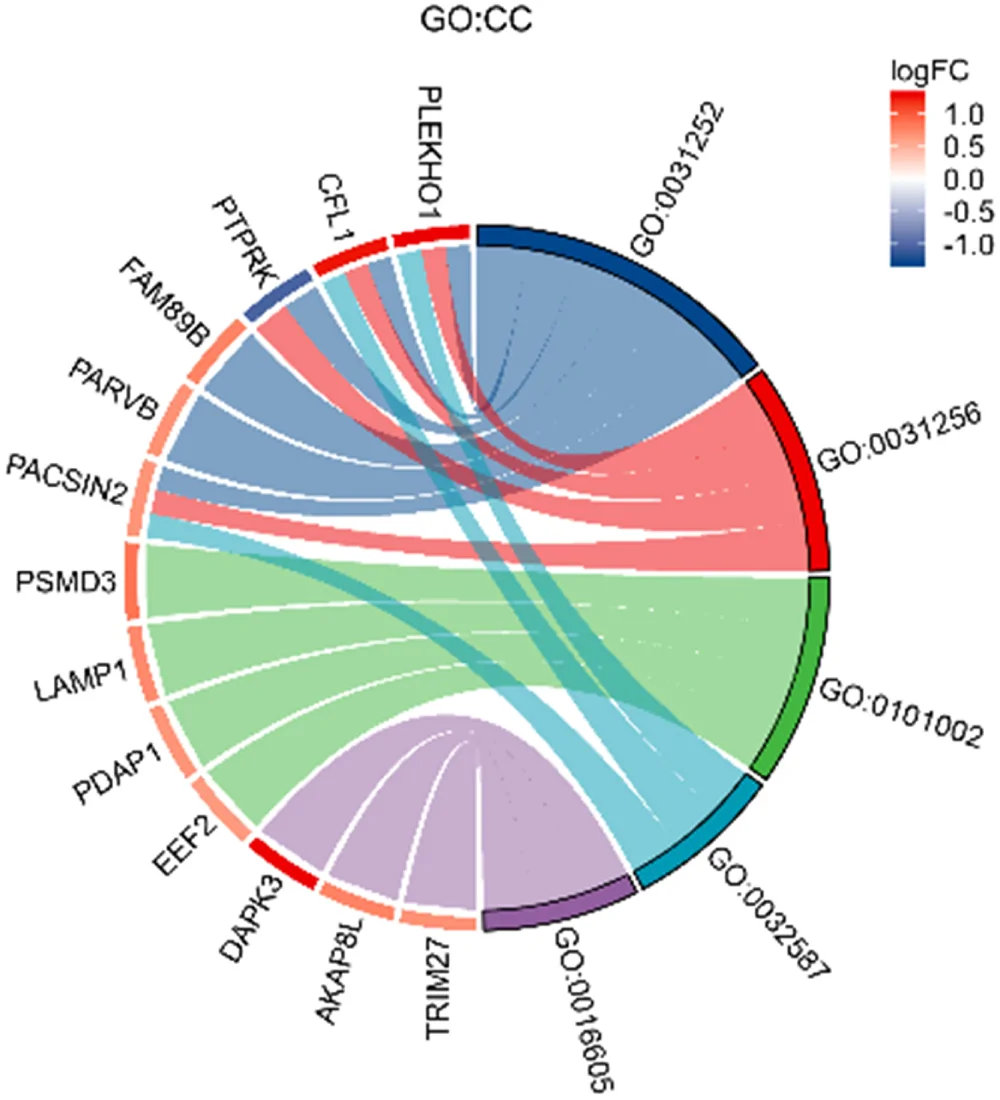
Chord diagram of candidate genes based on combined GO and KEGG functional enrichment analysis using log2 Fold Change (logFC) data.

Enrichment analysis revealed that candidate genes were predominantly associated with cellular components (CCs). These components specifically include the cell leading-edge, leading-edge membrane, ficolin-1-rich granule, ruffle membrane, and PML body (promyelocytic leukemia body), as shown in Table 1 and Figure 3.

**Table 1:** Enrichment analysis of candidate genes using GO and KEGG databases combined with log2 Fold Change(logFC).

Ontology	ID	Description	GeneRatio	BgRatio	*P* value	*P*.adj	*Q* value	geneID	Count	*Z* score
CC	G0-0031252	cellleadingedEE	6/51	416/19 594	0.001	0.082	0.069	PLEKHO1/CFL1/PTPRK/FAM89B/PARVB/PACSIN2	6	1.633
CC	GO:0031256	leadingedge membrane	4/51	175/19 594	0.001	0.082	0.069	PLEKHO1/CFL1/PTPRK/PACSIN2	4	1.000
CC	GO:0101002	ficolin-1-rich granule	4/51	185/19 594	0.001	0.082	0.069	PSMD3/LAMP1/PDAP1/EEF2	4	2.000
CC	GO:0032587	ruffle membrane	3/51	97/19 594	0.002	0.094	0.079	PLEKHO1/CFL1/PACSIN2	3	1.732
CC	GO:0016605	PML body	3/51	105/19 594	0.003	0.094	0.079	DAPK3/AKAP8L/TRIM27	3	1.732

### Analysis of candidate gene-protein interaction network and hub gene screening

**Figure 4.**
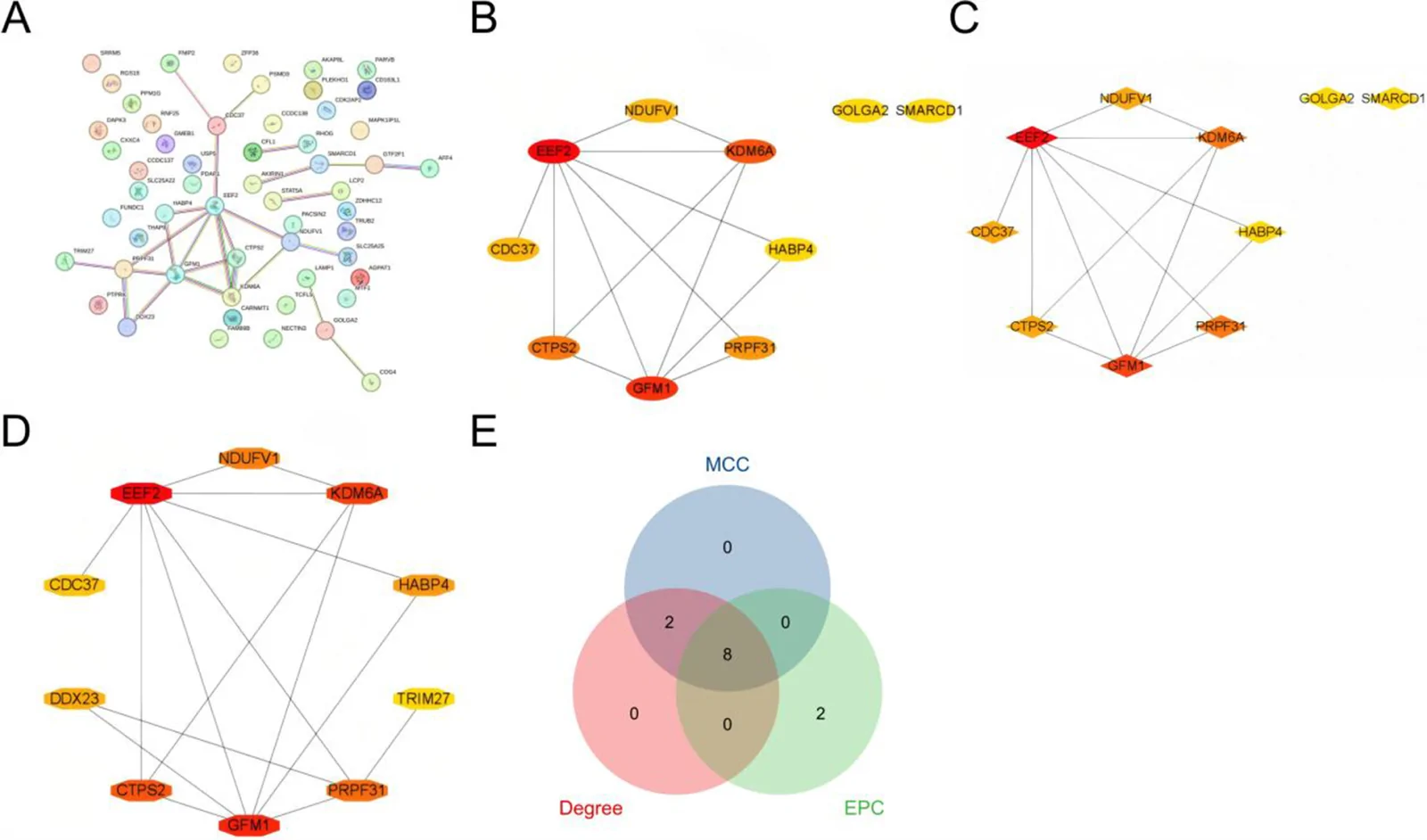
(A) Protein interaction network of candidate genes. (B) Top 10 genes, selected by the MCC algorithm. (C) Top 10 genes, selected by the Degree algorithm. (D) Top 10 genes, selected by the EPC algorithm. (E) Venn diagram showing the intersection of hub genes selected by the MCC, Degree, and EPC algorithms.

A total of 55 candidate genes were examined using the STRING database with a confidence score threshold >0.4. The analysis identified a network comprising 24 interacting proteins within the PPI network and 31 non-interacting proteins that did not interact with other proteins (Fig. 4A). To identify the top 10 genes, we employed three different methods using the CytoHubba plugin: MCC, Degree, and EPC. Tables 2–4 and Fig. 4B–4D present the results. The intersection of these methods identified hub genes EEF2, GFM1, KDM6A, CTPS2, PRPF31, NDUFV1, CDC37, and HABP4, as shown in Figure 4E.

**Table 2:** Presents the top 10 genes identified using the Maximal Clique Centrality (MCC) algorithm.

Order	Gene	MCC
1	EEF2	13
2	GFM1	12
3	KDM6A	8
4	CTPS2	6
5	PRPF31	5
6	NDUFV1	3
7	CDC37	3
8	GOLGA2	2
9	SMARCD1	2
10	HABP4	2

**Table 3:** The top 10 genes selected by the Degree algorithm.

Order	Gene	Degree
1	EEF2	7
2	GFM1	6
3	PRPF31	4
4	KDM6A	4
5	NDUFV1	3
6	CTPS2	3
7	CDC37	3
8	GOLGA2	2
9	SMARCD1	2
10	HABP4	2

**Table 4:** Top 10 genes selected by the EPC algorithm.

Order	Gene	EPC
1	EEF2	7.731
2	GFM1	7.524
3	KDM6A	7.249
4	CTPS2	7.025
5	PRPF31	6.895
6	NDUFV1	6.252
7	HABP4	6.035
8	DDX23	5.827
9	CDC37	5.261
10	TRIM27	4.138

### LASSO logistic regression analysis for diagnostic purposes

**Figure 5.**
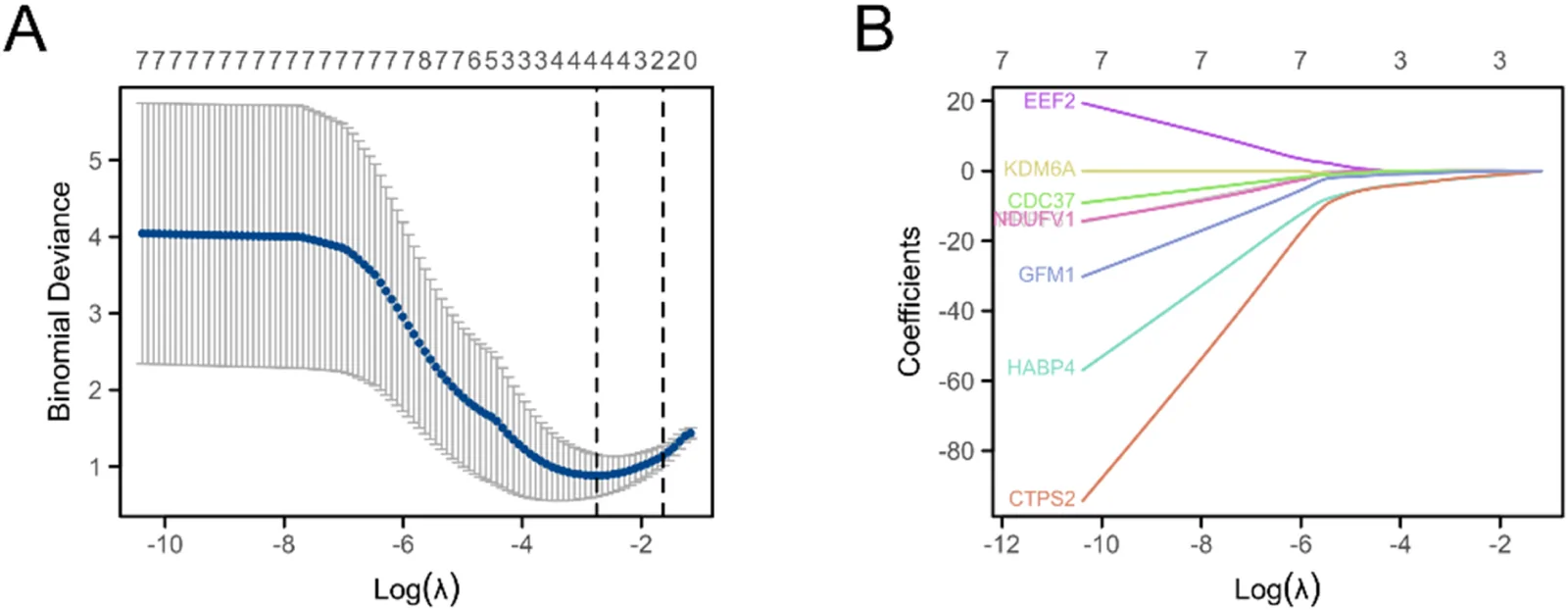
(A) Selection of variable coefficients in the LASSO logistic regression analysis for diagnosis based on hub gene. (B) Coefficient paths in the LASSO logistic regression analysis for diagnosis based on hub genes.

The results indicated that LASSO logistic regression for diagnostic purposes successfully identified PRPF31, HABP4, CTPS2, and GFM1 as hub genes. The *R*^2^ values obtained from the analysis of gene organization heterogeneity were 0.000, 0.009, 0.000, and 0.000. These genes are important in patients with PCOS and have been identified as potential biomarkers for diagnosing this condition. As illustrated in Figure 5A and 5B, these findings highlight their diagnostic value.

### Comparison of PCOS biomarker gene expression levels across different tissues: ROC analysis and validation of diagnostic performance

**Figure 6.**
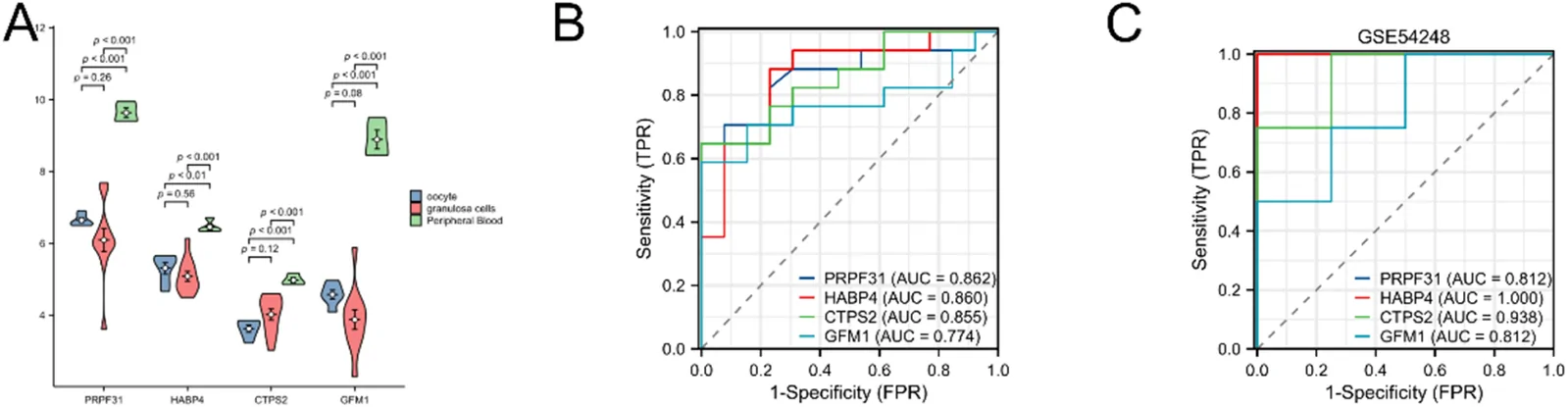
(A) Comparison of the expression levels of PRPF31, HABP4, CTPS2, and GFM1 in oocytes, granulosa cells, and peripheral blood samples. (B) Receiver operating characteristic (ROC) curve analysis assessing the diagnostic performance of PRPF31, HABP4, CTPS2, and GFM1 in the combined PCOS datasets. (C) Validation of the diagnostic accuracy of these genes in the GSE54248 dataset using ROC curve analysis.

PRPF31, HABP4, CTPS2, and GFM1 expression levels were significantly higher in peripheral blood than in oocytes and follicular granulosa cells (Fig. 6A). These genes demonstrated significant potential for diagnosing PCOS in patients; the AUC values were as follows: PRPF31, 0.862; HABP4, 0.860; CTPS2, 0.855; and GFM1, 0.774, as depicted in Figure 6B. Furthermore, validation analysis of the GSE54248 dataset confirmed that the expression of HABP4 and CTPS2 in peripheral blood had excellent diagnostic capabilities for PCOS, with AUC values of 1.000 and 0.938, respectively (Fig. 6C). The expression levels of PRPF31 and GFM1 in the peripheral blood demonstrated strong diagnostic capabilities, each with an AUC of 0.812, as shown in Figure 6C.

### Univariate and multivariate logistic regression analysis, along with nomogram construction

**Figure 7.**
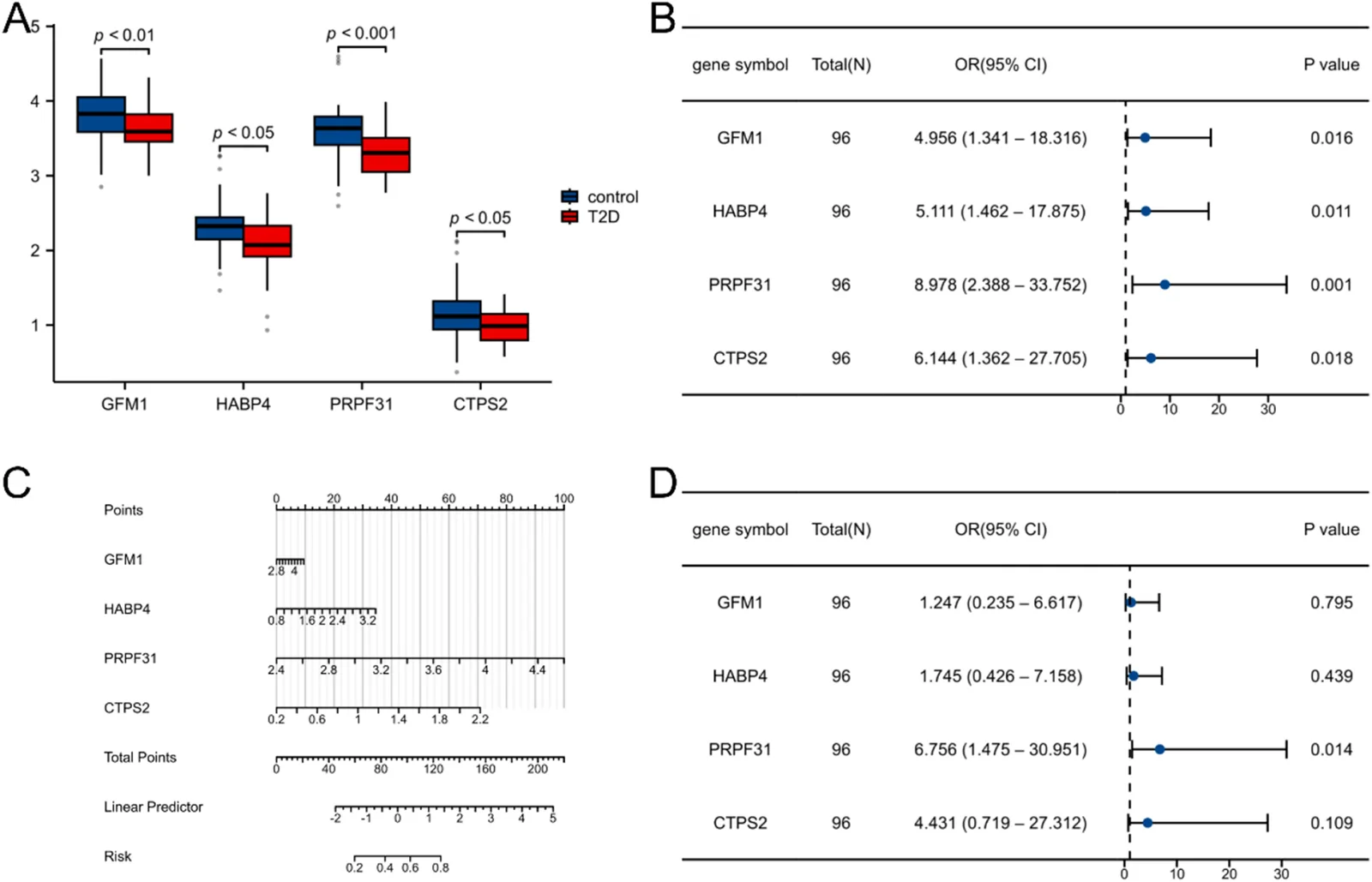
(A) Relative expression levels of PRPF31, HABP4, CTPS2, and GFM1 genes in the peripheral blood of T2D patients. (B) Univariate logistic regression analysis of these gene expression levels. (C) Diagnostic nomogram analysis based on these gene expression levels. (D) Multivariate logistic regression analysis of these gene expression levels.

Figure 7A illustrates that patients with T2D exhibited significantly reduced expression levels of PRPF31, HABP4, CTPS2, and GFM1 in peripheral blood compared to the control group. Univariate logistic regression analysis identified these genes (GFM1, HABP4, PRPF31, and CTPS2) as risk factors for T2D. A reduction in GFM1 expression correlated with a 3.956-fold heightened risk of T2D development, and a decrease in HABP4, PRPF31, and CTPS2 expression was associated with increased T2D risk by 4.111-fold, 7.978-fold, and 5.144-fold, respectively, as shown in Figure 7B. Multivariate logistic regression analysis identified PRPF31 as an independent risk factor for T2D, with reduced expression linked to a 5.756-fold higher risk, as illustrated in Figure 7D. The C-index results from the nomogram analysis suggest that this model has strong discriminatory power for predicting T2D, as illustrated in Figure 7C and Table 5.

**Table 5:** Presents the assessment of the diagnostic nomogram model utilizing GFM1, HABP4, PRPF31, and CTPS2 genes derived from peripheral blood samples of T2D patients.

Evaluation criteria	Evaluation content	Statistic	*P*-value
Model validation	Likelihood ratio test	Chi-square value: 18.104	0.0012
Discrimination evaluation	C index	C index: 0.747 (0.644–0.850)	1.63e-06
Calibration evaluation	Goodness of fit test	Chi-square value: 11.739	0.1632

## Discussion

PCOS is a hereditary disorder that affects many women and has substantial health implications. The precise cause of PCOS is unknown, but research suggests that it is a polygenic disorder influenced by epigenetic, developmental, and environmental factors. The complex nature of PCOS underscores the necessity for additional research to comprehensively understand its causes and impact on women’s health[19]. Research findings indicate that PCOS is closely linked to insulin resistance and metabolic syndrome^[^20,21^]^. The diverse symptoms associated with this condition can expose patients to a range of health risks, including infertility, diabetes, cardiovascular diseases, and mental health issues[1]. Research has consistently demonstrated a strong clinical link between PCOS and T2D, indicating potential shared underlying mechanisms. Consequently, this correlation highlights the importance of understanding the interconnected nature of these diseases, as evidenced by research findings^[^15,22^]^. Investigating the molecular mechanisms of PCOS and identifying related biomarkers is crucial for enhancing diagnostic accuracy and treatment efficacy for patients.

This study utilized machine learning techniques to integrate multi-chip data from various sources, including oocytes, granulosa cells, and peripheral blood. By conducting a comprehensive analysis of these data, we identified potential diagnostic biomarkers for PCOS and investigated their relationship with T2D. An innovative aspect of our approach is the cross-disease analytical perspective, which sheds light on the shared mechanisms between PCOS and T2D. By merging multiple gene expression microarray chip datasets, we identified 587 DEGs. These genes provide significant insights and enhance our understanding of the complex molecular mechanisms underlying PCOS. Their regulation is closely linked to the pathophysiological processes of PCOS, thereby providing a robust theoretical basis for the development of new diagnostic tools and treatment strategies.

In our analysis of the intersection between DEGs in PCOS and target genes associated with T2D, we identified 55 candidate genes, including PRPF31, HABP4, CTPS2, and GFM1. These genes may serve as promising biomarkers for PCOS. Identifying these biomarkers is crucial because they can enhance early diagnosis, facilitate timely treatment, and enable precise management of PCOS, which, in turn, significantly improves the effectiveness and applicability of clinical diagnosis and treatment. This discovery offers a fresh perspective and a novel approach for elucidating the intricate relationships between PCOS and T2D.

We conducted an analysis integrating log2 FC data with GO and the KEGG enrichment for our candidate genes. These findings indicate that these genes are predominantly associated with specific CCs, highlighting their potential significance in various biological processes. Therefore, it is essential to explore how the functions of these CCs influence the pathophysiological mechanisms of PCOS, particularly in critical areas such as cell signaling and metabolic regulation. Exploring the interactions of other biological pathways with these CCs could offer significant insights into the roles of these genes in PCOS pathogenesis. Such an understanding could significantly advance research in this field.

In our analysis of PPI networks, we identified eight key hub genes using three distinct methods: MCC, Degree, and EPC. The identified genes included EEF2, GFM1, KDM6A, CTPS2, PRPF31, NDUFV1, CDC37, and HABP4. Following this, we conducted LASSO regression on these hub genes, ultimately selecting four that are particularly significant for PCOS patients: PRPF31, HABP4, CTPS2, and GFM1. These genes not only contribute to biological research but also hold potential as crucial elements in future biomarker screening and the exploration of underlying mechanisms. Furthermore, a detailed examination of the interactions among these hub genes, particularly their regulatory roles in PCOS development, will offer significant insights into their contribution to disease progression. This understanding could pave the way for new clinical applications, establishing a robust foundation for diagnosis and treatment strategies in personalized medicine.

Analysis using the ROC curve reveals that the PRPF31, HABP4, CTPS2, and GFM1 genes serve as significant diagnostic markers for patients with PCOS. The expression levels of HABP4 and CTPS2 genes exhibit significant diagnostic sensitivity and specificity across different sample types. Our research indicates marked differences in the expression of PRPF31, HABP4, CTPS2, and GFM1 genes in oocytes and follicular granulosa cells compared with peripheral blood samples. This finding underscores the importance of evaluating these genes in different tissue types, such as oocytes, granulosa cells, and peripheral blood. The tissue-specific expression patterns of these genes provide critical insights for clinical applications, reinforcing the findings of previous studies that emphasize the significance of tissue specificity in disease diagnosis[23]. By comparing gene expression profiles from different tissue samples, we can deepen our understanding of the molecular mechanisms underlying PCOS, thereby offering robust support for future clinical practices.

A detailed analysis employing univariate and multivariate logistic regression was performed on PRPF31, HABP4, CTPS2, and GFM1 to assess their association with T2D risk. The univariate logistic regression results showed that decreased gene expression was associated with a higher risk of T2D. Multivariate logistic regression analysis identified PRPF31 as an independent risk factor for T2D, with reduced expression linked to a 5.76-fold increased risk of developing the disease, which is vital for evaluating T2D risk in patients with PCOS and greatly improves the precision of risk assessments. Additionally, these findings offer crucial insights into the early diagnosis and personalized treatment of patients with PCOS. By evaluating the concordance index (C-index) of the diagnostic model, we determined that it possessed a robust capacity to differentiate between patients with T2D. These findings support the potential application of this model in clinical practice.

Our study builds upon previous research that primarily focused on individual diseases by delving deeper into the intricate relationship between PCOS and T2D[24]. In contrast to previous research, we adopt a comprehensive approach by integrating multi-chip data – genomics, transcriptomics, and proteomics – to investigate the interactions between PCOS and T2D. This novel framework for cross-disease analysis consolidates data from multiple biological levels and provides new insights that may facilitate the creation of novel biomarkers and personalized treatment approaches.

The limitations of this study are mainly due to the small sample size, lack of experimental validation, and inadequate detailed analysis of clinical data. We integrated multiple datasets to identify DEGs; however, potential batch effects between these datasets could affect the reliability and generalizability of our results. Additionally, the lack of molecular validation using clinical samples meant that we could not confirm the practical applicability of our findings. Future research should aim to enhance the clinical relevance by expanding the sample size and utilizing diverse experimental methods to validate the findings.

This study systematically examined DEGs in PCOS patients, emphasizing their complex relationship with T2D. This research identifies a range of clinically significant biomarkers and provides solid groundwork for early diagnosis and targeted treatment approaches. These findings improve our understanding of the molecular mechanisms and biological processes associated with PCOS, as well as offer innovative ideas and directions for intervention strategies aimed at T2D and other related metabolic disorders. The implications of this study hold considerable promise for practical applications and long-lasting impacts in the field (Supplemental Digital Content Guidelines Flow Diagram, available at: http://links.lww.com/MS9/B258).

## Data Availability

The datasets generated and/or analyzed during the current study are available in the (GEO database) repository: https://www.ncbi.nlm.nih.gov/geo/.
